# Intracellular Accumulation and Secretion of YKL-40 (CHI3L1) in the Course of DMSO-Induced HL-60 Cell Differentiation

**DOI:** 10.3390/ph17040443

**Published:** 2024-03-29

**Authors:** Izabela Jatczak-Pawlik, Alicja Ewiak-Paszyńska, Małgorzata Domowicz, Anna Jurewicz, Mariusz Stasiołek

**Affiliations:** Department of Neurology, Medical University of Lodz, Kosciuszki Street 4, 90-419 Lodz, Poland; izabela.jatczak@umed.lodz.pl (I.J.-P.); alicja.ewiak-paszynska@umed.lodz.pl (A.E.-P.); malgorzata.domowicz@umed.lodz.pl (M.D.); anna.jurewicz@umed.lodz.pl (A.J.)

**Keywords:** cell differentiation, DMSO, YKL-40 (CHI3L1), HL-60 cells, neutrophil

## Abstract

YKL-40 (CHI3L1) is a matrix glycoprotein stored in human neutrophil-specific granules and released upon activation. While it is implicated in inflammation, cancer progression, and cell differentiation, its exact physiological role remains unclear. This study investigated the intracellular expression and secretion of YKL-40 by untreated and DMSO-treated HL-60 cells in association with surface expression of CD11b and CD66b throughout the differentiation process (up to 120 h). Secreted YKL-40 protein and mRNA levels of YKL-40, CD66b, and CD11b were measured by ELISA and quantitative RT-PCR, respectively. The intracellular YKL-40 and surface CD11b and CD66b expression were assessed by flow cytometry. A significant increase in CD11b expression confirmed DMSO-induced differentiation of HL-60 cells. Upon DMSO stimulation, YKL-40 mRNA expression increased in a time-dependent manner, unlike CD66b. The lack of CD66b (a granulocyte maturation and activation marker) on the surface of HL-60 cells might suggest that DMSO treatment did not induce full maturation or activation. The intracellular YKL-40 protein expression was increasing up to 96 h of DMSO treatment and then declined. YKL-40 secretion into the culture medium was detectable only at later time points (96 and 120 h), which was correlated with a decreased proliferation of DMSO-treated HL-60 cells. These findings suggest sequential changes in YKL-40 production and secretion during DMSO-induced differentiation of HL-60 cells and might contribute to a better understanding of YKL-40’s involvement in both physiological processes and disease development, including multiple sclerosis.

## 1. Introduction

Neutrophils are the predominant granulocyte population in peripheral blood. Granulopoiesis is a multi-step process in the bone marrow that leads to the differentiation of neutrophils from hematopoietic stem cells (HSCs) to, sequentially, myeloblasts, promyelocytes, myelocytes, metamyelocytes, band neutrophils, and, finally, mature segmented neutrophils. Cells in the first three stages of differentiation proliferate, while cells in the last three stages do not divide [1]. Neutrophils play a key role in orchestrating the inflammatory response. They produce reactive oxygen species (ROS) and release neutrophil extracellular traps (NETs), which are highly decondensed chromatin fibers decorated with antimicrobial proteins. Additionally, neutrophils release various enzymes from their granules. Depending on the protein contents, several types of granules were described including azurophilic or primary granules [containing, e.g., myeloperoxidase (MPO), neutrophil elastase (NE), CD63], secondary or specific granules [containing, e.g., lactoferrin, neutrophil gelatinase-associated protein lipocalin (NGAL), CD66b, YKL-40], and tertiary or gelatinase granules [containing, e.g., gelatinase and surface receptors including CD11b] [2,3]. Segmented neutrophils, the final differentiated cells, additionally form another type of granule named ficolin-1 granules (containing, e.g., actin and vanin-2) and produce secretory vesicles (SVs) containing actin, alkaline phosphatase, and certain receptors including CD11b and CD16 [2]. The azurophilic granules are formed first, followed by specific granules, gelatinase granules, and lastly, secretory vesicles in mature cells. Mature granulocytes release granules in the reverse order of their formation, starting with secretory vesicles and followed by others [4]. It is well established that neutrophil-derived granule proteins, along with cytokines, contribute to both the initiation and maintenance of inflammatory responses. While these mechanisms play a crucial role in defense against invading pathogens, they can also contribute to tissue damage and the development of various chronic inflammatory disorders, such as inflammatory bowel diseases, rheumatoid arthritis, chronic obstructive pulmonary disease, atherosclerosis, or multiple sclerosis [5].

YKL-40 is a glycoprotein, also known as human chitinase-3-like-1 protein (CHI3L1) involved in immunological responses [6]. The level of YKL-40 in serum, synovial fluid, or cartilage has been extensively investigated in various inflammatory disorders, including thyroid disease, chronic obstructive pulmonary disease, diabetes, cardiovascular disease, multiple sclerosis, and rheumatoid arthritis [7]. Additionally, upregulation of this glycoprotein has been observed in several types of solid tumors (colon, breast, prostate, ovarian, and glioblastoma) [7]. Based on these findings, YKL-40 might be considered a potentially useful factor in the clinical diagnosis and prognosis of various diseases. While the precise role of YKL-40 in the disease is not fully understood, the pattern of its expression suggests potential involvement in cell proliferation and differentiation, inflammation, tissue remodeling, protection against apoptosis, stimulation of angiogenesis, and tumor metastasis [8]. In contrast, there is little information about the role of YKL-40 in the physiological processes. High YKL-40 expression has been reported during early human development [9], leading to the suggestion that this glycoprotein might be important for the initial differentiation of human embryonic stem cells (hESCs) towards the ectoderm and neuroectoderm [10]. However, the exact physiological function of YKL-40 in embryonic development as well as in cell homeostasis remains largely unknown.

Since Volck et al. showed that activated neutrophils from patients with active rheumatoid arthritis secreted YKL-40 into synovial fluid [3]; neutrophils became the next cell producer of YKL-40 in inflammatory conditions as macrophages, chondrocytes, synovial cells, and various tumor cells [11]. Moreover, the presence of YKL-40 within specific neutrophil granules of healthy subjects has been demonstrated by its colocalization with lactoferrin, a marker for specific neutrophil granules [3].

The HL-60 cell line, derived from the peripheral blood leukocytes of a female patient with acute promyelocytic leukemia [12] can differentiate in response to various stimuli, including dimethyl sulfoxide (DMSO), into neutrophil-like cells [13]. Although the mechanism underlying DMSO-induced differentiation of HL-60 cells remains not fully understood and demands further investigation, it is widely accepted as an experimental model [14,15] for studying various processes in normal peripheral blood granulocytes, including phagocytosis [16], chemotaxis [16,17], or rolling interactions [18].

The intracellular expression and secretion of YKL-40 in association with the surface expression of CD11b/Integrin alpha M and CD66b/CEACAM-8 in undifferentiated and DMSO-differentiated HL-60 cells were investigated in the present study. CD66b, a granulocyte maturation and activation marker, was chosen because of its colocalization in specific granules of peripheral blood neutrophils with YKL-40 [2,3]. CD11b integrin, a previously reported marker of cell differentiation, was selected to determine the degree of HL-60 cell differentiation in response to DMSO [19,20].

## 2. Results

### 2.1. Basal mRNA Expression of YKL-40, CD11b, and CD66b in HL-60 Cells

The mRNA expression levels of the glycoprotein YKL-40 and adhesion molecules CD11b and CD66b in undifferentiated HL-60 cells were measured using real-time RT-PCR. Expression levels were normalized to the reference gene TBP (copies per 1000 TBP mRNA).

**Figure 1:**
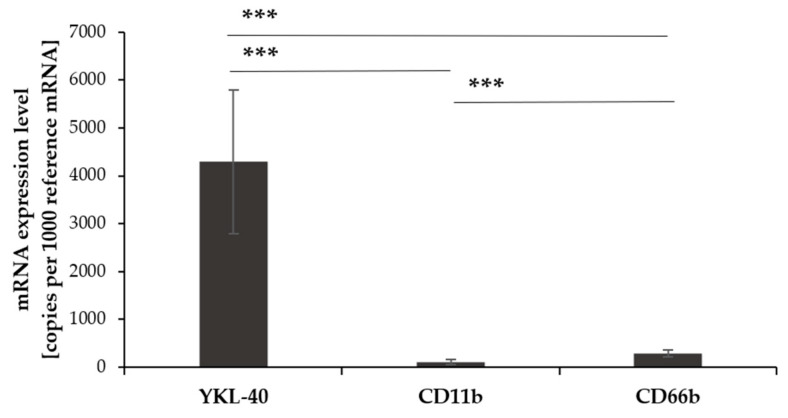
The mRNA expression of YKL-40, CD11b, and CD66b in undifferentiated HL-60 cells. Real-time RT-PCR was used to measure mRNA levels, normalized to the reference gene TBP (mean ± SD, *n* = 10), ***, *p* < 0.0005.

YKL-40 mRNA expression in undifferentiated HL-60 cells was significantly higher than CD11b and CD66b mRNA (31-fold and 21.7-fold difference, respectively, *p* < 0.0005). CD66b mRNA expression was significantly higher than CD11b (1.44-fold difference, *p* < 0.0005) (Figure 1).

### 2.2. Expression of YKL-40, CD11b, and CD66b during DMSO-Induced HL-60 Cells Differentiation

**Figure 2:**
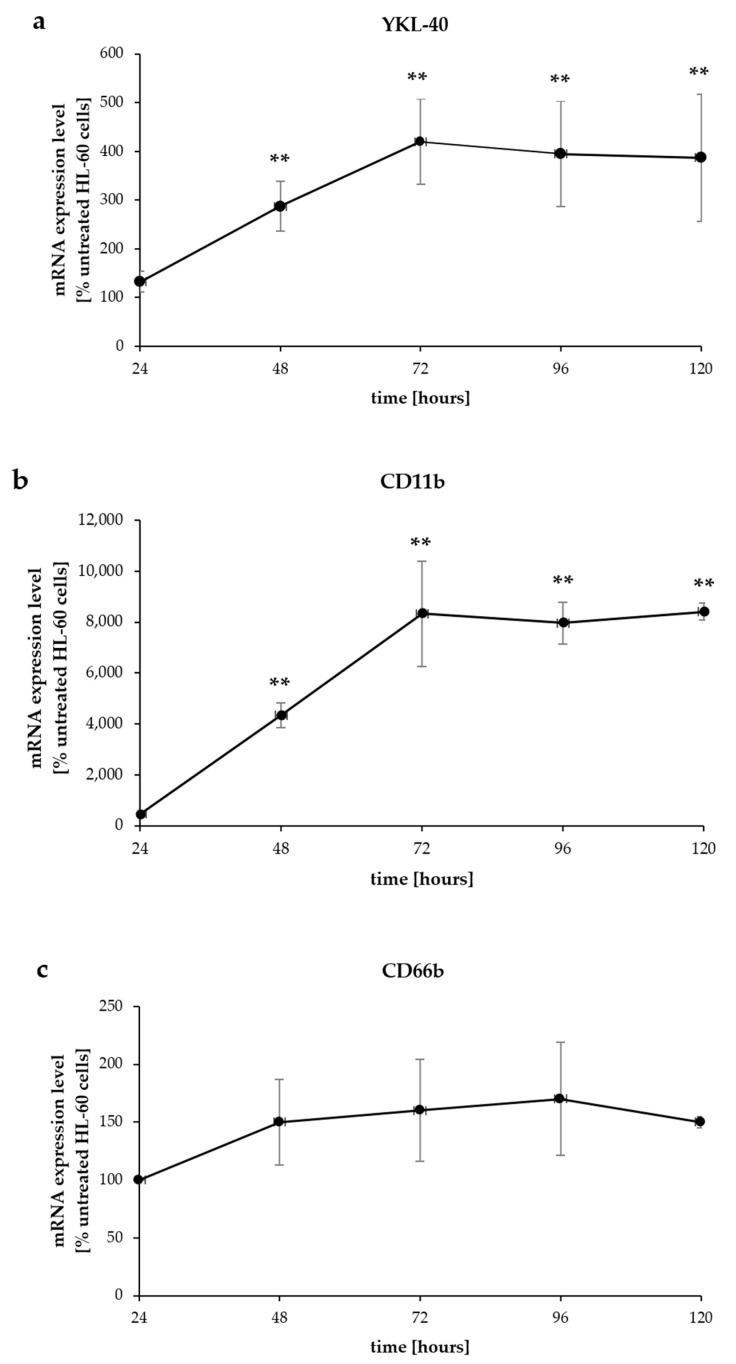
The kinetics of YKL-40 (**a**), CD11b (**b**), and CD66b (**c**) mRNA expression in HL-60 cells upon 1% DMSO stimulation. HL-60 cells were stimulated with DMSO for 0, 24, 48, 72, 96, and 120 h. The mRNA expression levels of YKL-40, CD11b, and CD66b were measured by RT-PCR and normalized to the reference gene TBP. Data are presented as the percentage of values of undifferentiated cells (time 0). YKL-40 and CD11b mRNA expression showed a significant increase (**, *p* < 0.005) at time points 48, 72, 96, and 120 h compared to 24 h of DMSO stimulation. Data are mean ± SD (*n* = 3).

Following 1% DMSO-induced differentiation of HL-60 cells, the mRNA expression of YKL-40 and CD11b was assessed at five time points (24, 48, 72, 96, and 120 h). The mRNA levels were normalized to the mRNA levels in undifferentiated HL-60 cells (time 0). YKL-40 and CD11b mRNA expression showed a significant increase (*p* < 0.005) at time points of 48, 72, 96, and 120 h compared to 24 h of DMSO stimulation (Figure 2a,b). Both YKL-40 and CD11b mRNA displayed similar time-dependent expression patterns, with a gradual increase in expression within 72 h, reaching a plateau at 96 h, and remaining at these levels up to 120 h.

**Figure 3:**
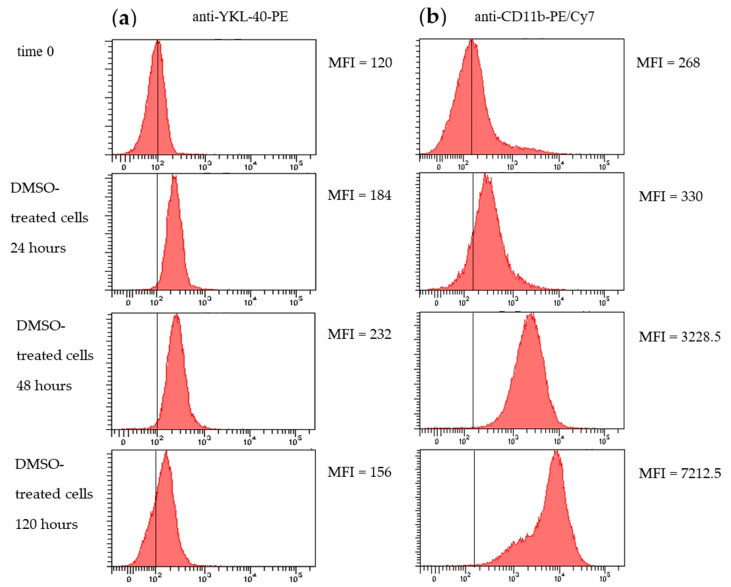
YKL-40 (intracellular) (**a**) and CD11b (surface) (**b**) protein expression. HL-60 cells untreated or treated with 1% DMSO for 24, 48, and 120 h were stained for YKL-40 (**a**) and CD11b (**b**) to assess protein expression using flow cytometry. The histograms are representative of three independent experiments. The arithmetic mean fluorescence intensity (MFI) for YKL-40 (**a**) and CD11b (**b**) staining is shown on the side of each histogram.

**Figure 4:**
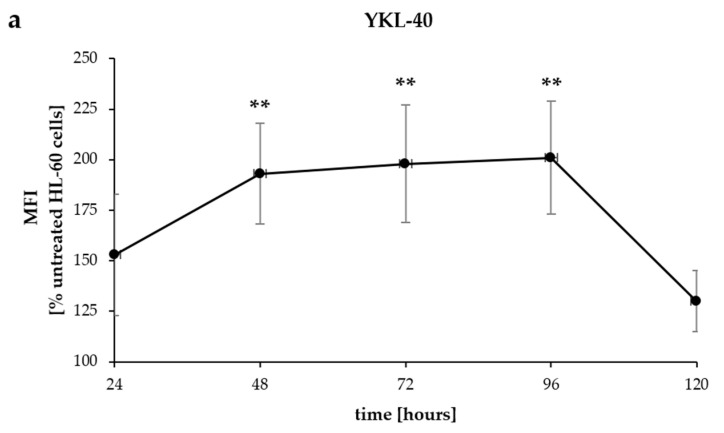

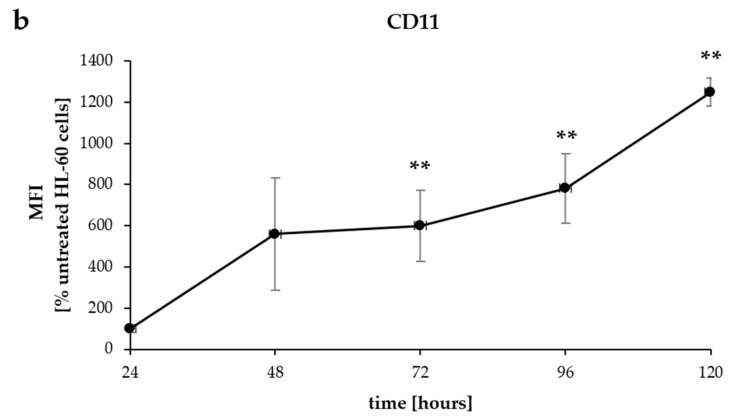
The kinetics of protein expression of YKL-40 (**a**) and CD11b (**b**) in HL-60 cells upon 1% DMSO stimulation. HL-60 cells were stimulated with DMSO for 0, 24, 48, 72, 96, and 120 h. YKL-40 and CD11b protein expression was measured by flow cytometry, and levels were quantified as the arithmetic mean fluorescence intensity (MFI) and presented as the percentage of MFI values measured for undifferentiated cells (time 0). YKL-40 protein expression significantly decreased at 120 h compared to time points of 48, 72, and 96 h (**, *p* < 0.005). CD11b protein expression showed a significant increase at 72, 96, and 120 h compared to 24 h (**, *p* < 0.005). Data are mean ± SD (*n* = 3).

Since mRNA levels may not reflect protein expression, we assessed the intracellular expression of YKL-40 and the surface expression of CD11b by flow cytometry. Both YKL-40 and CD11b protein levels showed a continuous increase up to 96 h, with respective 2-fold and 7.8-fold increases at this time point (Figure 3a,b). After the initial enhanced production, intracellular YKL-40 protein levels significantly decreased (*p* < 0.005) at 120 h compared to previous time points (48, 72, and 96 h) and reached a level below those observed in undifferentiated cells, representing a 1.3-fold reduction (Figure 4a,b). In contrast, surface CD11b protein expression continued to increase throughout differentiation, reaching a 12.5-fold increase at 120 h compared to 24 h of DMSO stimulation (Figure 3a,b).

CD66b mRNA expression showed a slight increase upon DMSO-induced differentiation but this was not statistically significant (Figure 2c). Similarly, protein expression of CD66b did not show any significant changes as compared to untreated cells.

#### 2.2.1. Viability of DMSO-Treated HL-60 Cells

Three assays were used to assess the cell viability after 1% DMSO treatment: (1) propidium iodide (PI) staining and flow cytometry to detect the subdiploid peak revealing DNA fragmentation indicative of cell apoptosis, (2) annexin V/PI staining and flow cytometry analysis to detect apoptotic and necrotic cells, and (3) cell counting using the TC20 Automated Cell Counter after dead cell exclusion with Trypan Blue staining.

**Figure 5:**
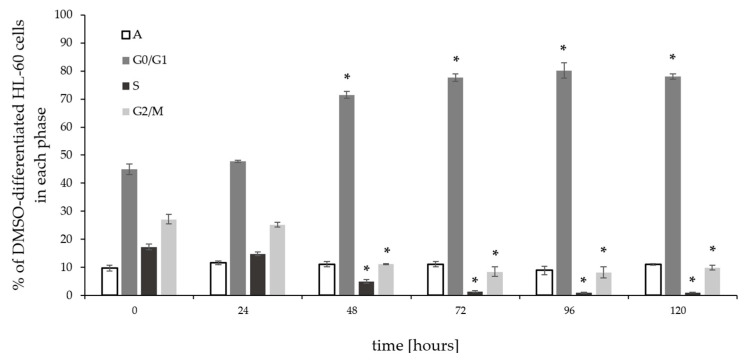
DMSO-induced cell cycle arrest in HL-60 cells. HL-60 cells were treated with 1% DMSO for 0, 24, 48, 72, 96, and 120 h. The cell cycle and changes in DNA content were assessed by flow cytometry using propidium iodide (PI) staining. The differences in the distribution of cells into all cell cycle phases were statistically significant (*, *p* < 0.05) as compared to untreated cells (time 0). The percentage of apoptotic cells remained constant. Data are mean ± SD (*n* = 3).

**Figure 6:**
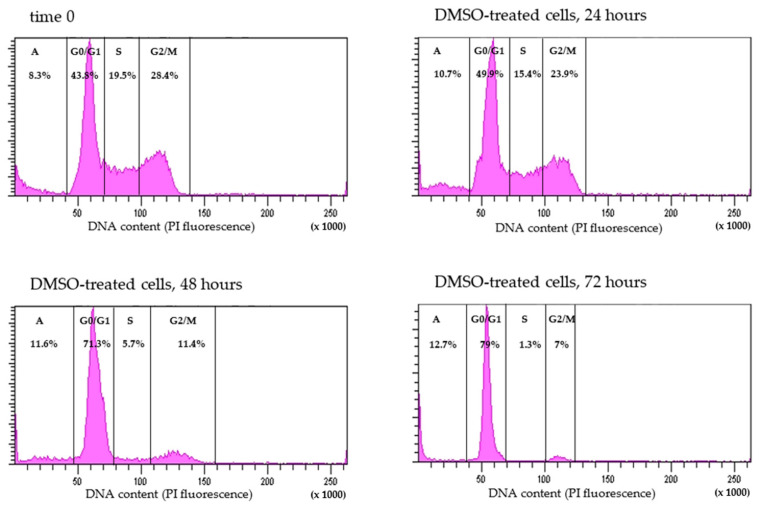

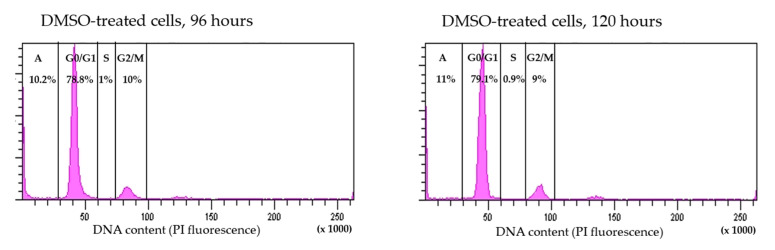
Exemplary analysis of HL-60 cell cycle after DMSO treatment. HL-60 cells were treated with 1% DMSO for 0, 24, 48, 72, 96, and 120 h, stained with propidium iodide (PI), and analyzed by flow cytometry to assess the cell cycle by changes in DNA content. Cells with DNA content to the left of the G0/G1 phase were identified as apoptotic (A). The percentage of cells in each phase (A, G0/G1, S, G2/M) has been shown on each histogram. The histograms are representative of three independent experiments.

PI staining allowing the assessment of dead cells by measurement of low molecular weight DNA fragments (Figure 5 and Figure 6) and Trypan Blue staining used for the exclusion of dead cells gave a similar proportion of living cells, 92% and 95% at all time points for both assays, respectively. The annexin V/PI assay indicated a slightly lower survival rate of approximately 85% due to detecting early apoptotic cells. These results suggest that DMSO treatment did not significantly affect HL-60 cell survival up to 120 h.

#### 2.2.2. Effect of DMSO Treatment on HL-60 Cell Proliferation

To investigate the effect of DMSO on cell proliferation and the cell cycle, HL-60 cells were treated with 1% DMSO for 24, 48, 72, 96, and 120 h, stained with PI for DNA cell content, and then analyzed by flow cytometry. The percentage of the HL-60 cells in the G0/G1 phase of the cell cycle increased after 48 h of DMSO stimulation and was high since then up to 120 h, as compared to untreated HL-60 cells (45%) and cells at 24 h of DMSO-induced differentiation (48%) (Figure 5 and Figure 6). This G0/G1 phase arrest was confirmed by a decrease in the percentage of cells in the S and G2/M phases of the cell cycle reflecting cell proliferation inhibition. Exposure of exponentially growing HL-60 cells with DMSO resulted in a marked decrease of the percentage of cells in the S-phase from 17% (untreated HL-60 cells, time 0), 15% at 24 h, and 5% at 48 h to 1% at 72, 96, and 120 h, confirming inhibition of cell proliferation. Similarly, a significant decrease in the percentage of cells in the G2/M-phase was observed from 27% in untreated HL-60 cells to 25% at 24 h, 11% at 48 h, and 9% at 72, 96, and 120 h of stimulation with DMSO. These data suggest that DMSO inhibited the proliferation of HL-60 cells at the G0/G1 phase.

**Figure 7:**
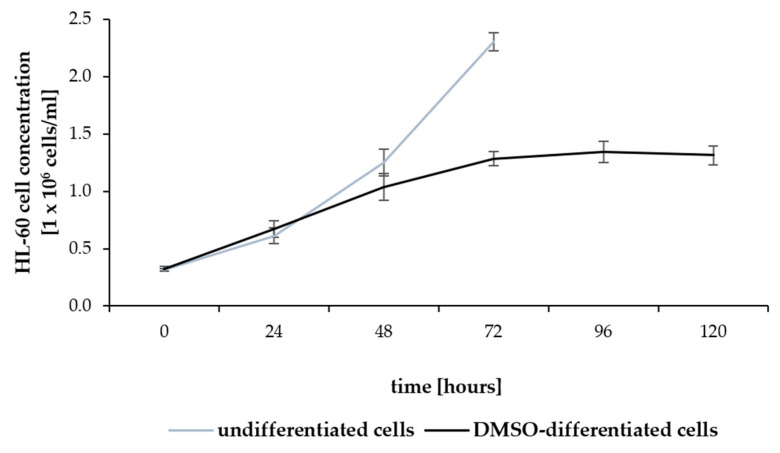
HL-60 cell growth inhibition after DMSO treatment. The concentration of viable HL-60 cells cultured with or without 1% DMSO was counted with a TC20 Automated Cell Counter after the exclusion of dead cells by Trypan Blue staining. Data are mean ± SD (*n* = 3).

Trypan Blue staining revealed a moderate reduction in the concentration of viable HL-60 cells after 48 h of DMSO treatment compared to the exponential growth observed in untreated cells. By 72 h of differentiation, the live cell concentration in DMSO-treated HL-60 cells was half that of the untreated cells. After that time point, the concentration of DMSO-stimulated HL-60 cells remained stable till the end of the experiment (Figure 7). This stabilization of HL-60 cell concentration was not accompanied by an increase in dead cell number, suggesting inhibition of cell proliferation by DMSO rather than cytotoxicity. Compared to the untreated cells, DMSO treatment resulted in a significant reduction in the proliferation rate of HL-60 cells, with a decrease of 44% and 47% observed at 48 and 72 h, respectively (Figure 7).

#### 2.2.3. YKL-40 Secretion by DMSO-Differentiated HL-60 Cells

**Figure 8:**
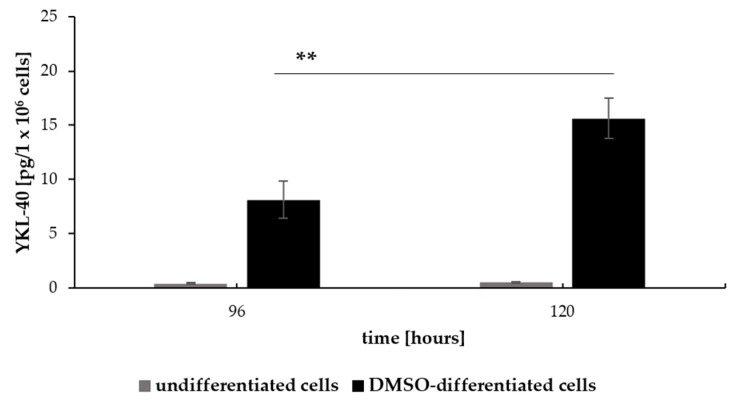
YKL-40 secretion from HL-60 cells after DMSO treatment. YKL-40 protein levels were measured at 96 and 120 h by ELISA in culture supernatants of undifferentiated and 1% DMSO-differentiated HL-60 cells. The data present the amount of YKL-40 secreted per 1 × 10^6^ cells in culture (mean ± SD, *n* = 3). **, *p* < 0.005.

We determined the effect of DMSO-induced differentiation of HL-60 cells on YKL-40 production and secretion. YKL-40 concentration was measured by ELISA at all time points (0, 24, 48, 72, 96, and 120 h), and the secreted amount of YKL-40 was calculated on 1 × 10^6^ cells. YKL-40 was undetectable in the culture supernatants of undifferentiated cells (0 h) or cells differentiated for 24, 48, and 72 h. DMSO-treated HL-60 cells began to secrete detectable levels of YKL-40 into the culture supernatant since 96 h. This secretion further increased at 120 h, correlating with a decrease in intracellular YKL-40 protein expression detected by flow cytometry. Importantly, YKL-40 secretion by DMSO-differentiated HL-60 cells increased nearly 2-fold at 120 h compared to 96 h (*p* < 0.005) (Figure 8). YKL-40 protein was undetectable in the culture medium of undifferentiated HL-60 cells at 96 and 120 h.

## 3. Discussion

The neutrophils derived from peripheral blood contain azurophilic, specific, gelatinase granules, and secretory vesicles [21], unlike terminally differentiated HL-60 cells. DMSO-differentiated HL-60 cells contain only azurophilic granules and lack the specific granules [22,23] and other late-stage granules [24,25] typical for granulocytic maturation. The experiments performed so far demonstrated that HL-60 cells fail to express specific granule proteins like lactoferrin and neutrophil gelatinase-associated lipocalin (NGAL) even after differentiation into neutrophil-like cells [26,27,28]. Moreover, the lactoferrin gene was not transcribed in DMSO-stimulated HL-60 cells, suggesting a defect in production or regulation [26,27]. Similarly, NGAL did not show expression at both mRNA and protein levels in the HL-60 cells [28]. However, these cells stably transfected with NGAL cDNA could synthesize NGAL protein, indicating functional synthesis pathways [28]. Taking into account the above mentioned findings, we evaluated the intracellular expression and potential ability of YKL-40 secretion in both undifferentiated and DMSO-differentiated HL-60 cells (up to 120 h) in correlation with the surface expression of CD11b and CD66b. Surface marker CD11b was selected to determine the degree of HL-60 cell differentiation in response to DMSO stimulation [19,20]. CD11b is localized in specific and gelatinase granules [29] and is considered a well-established surface marker for differentiated neutrophils [30]. CD66b, a marker for granulocyte maturation, is stored intracellularly within specific granules as YKL-40 and, upon cell activation, is translocated to cell surface [2]. Therefore, CD66b was selected as a secondary marker to CD11b for monitoring HL-60 cell differentiation [31]. The expression levels of CD11b and CD66b detected in our experiment, in both undifferentiated and DMSO-differentiated cells, were consistent with previous reports [31,32,33]. Unstimulated HL-60 cells had a low expression of both CD11b and CD66b. As shown by other groups, CD11b expression was gradually increasing following DMSO stimulation in our experiments [34,35], indicating differentiation process. However, CD66b remained unchanged over the 120 h DMSO stimulation, suggesting that such conditions did not allow for end-stage cell maturation. Importantly, CD66b expression in HL-60 cells might be induced in HL-60 cells by stimuli other than DMSO. Veselská et al. showed that a combined treatment with caffeic acid (CA) and all-trans retinoic acid (ATRA) significantly increased the surface expression of CD66b on HL-60 cells [31]. In another study, it was revealed that CD66b surface expression on mature granulocytes requires activation by additional stimulation with, e.g., N-formylmethionyl-leucyl-phenylalanine (fMLF), lipopolysaccharide (LPS), and complement component 5a (C5a) [36].

Our study demonstrated a link between YKL-40 production and secretion and the differentiation process of HL-60 cells. YKL-40 protein expression increased in parallel to CD11b in a time-dependent manner, reaching its highest level at 96 h. After this time point, intracellular YKL-40 protein levels began to decline, while CD11b protein expression continued to increase. Importantly, the YKL40 protein reached the detectable level in culture medium at 96 h of DMSO stimulation, and its concentration increased at the 120 h time point. These findings suggest that YKL-40 intracellular accumulation correlates with the early stages of differentiation, followed by its secretion from differentiated, non-proliferating HL-60 cells. DMSO-induced inhibition of HL-60 cell proliferation stays in line with the fact that mature myeloid cells [37] lose their proliferative capacity upon differentiation [38]. Thus, we propose that enhanced production of YKL-40 might be correlated with DMSO-induced HL-60 cell differentiation and potentially serve as another molecular marker for the initial phase of maturation. Nonetheless, CD66b surface expression was not detected on HL-60 cells despite the induction of YKL-40 production and secretion. The mechanisms underlying this observation are not clear and require further studies.

Finally, our work has demonstrated that the lack of specific and gelatinase granules in HL-60 cells did not prevent the expression and secretion of proteins typically stored within these granules. This finding suggests the existence of alternative pathways for storage and secretion of these proteins [39].

In conclusion, our study provides, for the first time, evidence that DMSO differentiation of HL-60 cells is correlated with the synthesis and secretion of YKL-40 (CHI3L1). Understanding the regulation of YKL-40 in these cells, and potentially in neutrophils and their bone marrow precursors, might be of importance in the development of new strategies for managing chronic inflammatory diseases. As the present study did not identify a specific regulation of YKL-40 synthesis and secretion, further studies involving functional experiments are necessary to gain deeper insights into the mechanism regulating YKL-40 expression in HL-60 cells.

## 4. Materials and Methods

### 4.1. Cell Culture and Differentiation

The HL-60 cell line was purchased from ATCC (Manassas, VA, USA). Cells were cultured in RPMI-1640 medium supplemented with 15% heat-inactivated fetal bovine serum (FBS) in a humidified atmosphere containing 5% CO_2_ at 37 °C. Cells were passaged three times per week to a density of 3 × 10^5^ cells/mL and growth to confluence at 1–2 × 10^6^ cells/mL. Only cells cultured for no longer than 6 weeks were used for the experiments. To induce differentiation, viable log-phase cells were resuspended in complete RPMI-1640 growth medium with 15% FBS at a density of 3 × 10^5^ cells/mL containing 1% dimethyl sulfoxide (DMSO, Sigma-Aldrich, St. Louis, MO, USA) and incubated within 120 h. Based on the previously published results where cells were differentiated but not yet dying, we chose 120 h of observation as optimal for the assessment of DMSO-induced differentiation [40]. Since DMSO is more viscous, a premix medium containing 1% DMSO was prepared before adding cells. To prepare cells for each experiment, cultures were centrifuged and washed twice in phosphate-buffered saline (PBS) at time points 24, 48, 72, 96, and 120 h. Untreated HL-60 cells served as the control at time 0 for each experiment. The cell viability was quantified daily by Trypan Blue dye exclusion with a TC20 Automated Cell Counter (Bio-Rad, Hercules, CA, USA). Additionally, a FITC Annexin V Apoptosis detection kit (BD Pharmingen, Franklin Lakes, NJ, USA) was used to determine the viability of differentiated HL-60 cells using the BD LSR II flow cytometer (BD Biosciences, Franklin Lakes, NJ, USA). The assay was performed strictly according to the manufacturer’s protocol.

### 4.2. Cell Cycle Analysis

The cell cycle was assessed by flow cytometry and propidium iodide (PI) staining. HL-60 cells (2 × 10^6^) were washed twice with ice cold PBS and resuspended in 1 mL of PBS. Fixation was achieved by incubating the cells with 80% ethanol (1 mL) on ice for 90 min. Following fixation, the cells were washed with PBS and resuspended in 1 mL of PBS containing 1 µL RNAse type II-A (5 mg/mL, Sigma-Aldrich) for RNA removal. Propidium iodide staining was performed at 37 °C for 30 min with 5 µL of PI solution (1 mg/mL, Sigma-Aldrich). The DNA content of 15,000 events was measured using the BD LSR II flow cytometer (BD Biosciences). The fluorescence intensity data was then analyzed to quantify the percentage of cells in the A (apoptotic phase), G0/G1, S, and G2/M-phases of the cell cycle.

### 4.3. Flow Cytometric Analysis

The expression of intracellular YKL-40 and membrane proteins CD66b and CD11b in untreated and 1% DMSO-treated cells was detected using the BD LSR II flow cytometer (BD Biosciences). Monoclonal antibodies specific for surface proteins anti-CD11b-PE/Cy7 (clone ICRF44, BD Biosciences) and anti-CD66b-APC (clone G10F5, BD Biosciences) were used for staining, following the manufacturer’s recommendations. For the intracellular protein, an anti-CHI3L1 (YKL-40)-PE polyclonal antibody (Bioss Antibodies, Boston, MA, USA) was used for staining at the experimentally determined concentration of 0.25 µg/mL. HL-60 cells (2 × 10^6^ cells) were washed twice with PBS, resuspended in 50 µL of blocking buffer (4% FBS and 2% human serum IgG from Sigma-Aldrich in PBS), and incubated with the monoclonal surface antibodies for 30 min at 4 °C. After washing with PBS, the cells were fixed with 2% paraformaldehyde (200 µL) for 10 min at 37 °C, followed by permeabilization with 70% ethanol (200 µL) on ice for 30 min. Permeabilized cells were then resuspended in 50 µL of blocking buffer and incubated for 30 min on ice. Subsequently, the cells were incubated with the anti-YKL-40-PE antibody for 20 min on ice and finally resuspended in 0.5 mL of PBS. The cells were protected from light at all stages of staining. The Live/Dead fixable dead cell stain kit (Thermo Fisher Scientific, Waltham, MA, USA) was used to determine the viability of the untreated and DMSO-treated HL-60 cells after fixation. The samples were analyzed using the same settings for forward and side scatter throughout the experiments. Compensation was performed to remove any spectral overlap between the fluorophores PE, PE-Cy7, and APC. The expression of the analyzed molecules (CD11b, CD66b, and YKL-40) was presented as the arithmetic mean fluorescence intensity (MFI). To exclude autofluorescence, MFI was also measured for untreated and DMSO-treated but unlabeled HL-60 cells before the experiments. Appropriate isotype controls were used. The results were obtained from three independent experiments.

### 4.4. Gene Expression Assay

The gene expression level was determined by a quantitative real-time reverse transcription-polymerase chain reaction (RT-PCR) in 96-well plates on an Applied Biosystems 7500 Real-time PCR System. Total cellular RNA was isolated using TRI Reagent (Merck, St. Louis, MO, USA) according to the manufacturer’s protocol. Complementary DNA (cDNA) was transcribed from mRNA using a High Capacity cDNA Reverse Transcription Kit (Thermo Fisher Scientific) and used for RT-PCR amplification with an RT HS PCR Mix SYBR (A&A Biotechnology) according to the manufacturer’s protocol, with 0.25 μM concentration of forward and reverse intron-spanning primers. The primer sequences were as follows: YKL-40 (CHI3L1), forward (fw) 5′-CGTCAACACACTCAAGAACAGG-3′, reverse (rv) 5′-TCTTGGAAAATCTTTGAGACCCA-3′, CD11b, fw 5′-CAGCTTCAGGGATCCAGGG-3′, rv 5′-GGCCCAGGGACATGTTCA-3′, CD66b, fw 5′-AAATGGTCAGAGTCTCCCGG-3′, rv 5′-TCTGAAGGGGAAATGGTGGG-3′ and TBP, fw 5′- CACGAACCACGGCACTGATT-3′, rv 5′-TTTTCTTGCTGCCAGTCTGGAC-3′. Expression levels were determined as the number of target mRNA copies normalized to 1000 copies of the reference gene TBP (TATA-box binding protein). All samples were run in duplicate, and the experiment was repeated three times. The data represent a mean from three independent experiments.

### 4.5. YKL-40 Secretion Assay

YKL-40 protein concentration was measured by a commercially available ELISA kit from Wuhan EIAab Science (Wuhan, China). The assay was performed strictly following the manufacturer’s instructions, with absorbance measured at 450 nm using an Epoch plate reader (BioTek, Winooski, VT, USA). Supernatants of DMSO-differentiated HL-60 cells at each time point (0, 24, 48, 72, 96, and 120 h) were collected and stored at −80 °C for further use. The data, derived from three independent experiments, represent the mean YKL-40 secretion per 1 × 10^6^ cells.

### 4.6. Statistical Analysis

ANOVA followed by Tukey’s post-hoc test was applied. Asterisks denote the level of statistical significance (*, *p* < 0.05; **, *p* < 0.005; ***, *p* < 0.0005). Data are presented as arithmetic mean ± standard deviation (SD). Statistical analysis was performed using the Statistica v.13 software (TIBCO Software Inc., Palo Alto, CA, USA).

## Data Availability

Data are contained within the article.
